# Comprehensive Analyses of Breads Supplemented with Tannic Acids

**DOI:** 10.3390/foods12203756

**Published:** 2023-10-12

**Authors:** Yanbin Guan, Xun Yang, Chuang Pan, Jie Kong, Ruizhe Wu, Xueli Liu, Yuesheng Wang, Mingjie Chen, Miao Li, Qiong Wang, Guangyuan He, Guangxiao Yang, Junli Chang, Yin Li, Yaqiong Wang

**Affiliations:** 1 The Genetic Engineering International Cooperation Base of Chinese Ministry of Science and Technology, The Key Laboratory of Molecular Biophysics of Chinese Ministry of Education, College of Life Science and Technology, Huazhong University of Science & Technology, Wuhan 430074, China; gyb@hust.edu.cn (Y.G.); u202112936@hust.edu.cn (X.Y.); d202280833@hust.edu.cn (C.P.); u202112929@hust.edu.cn (J.K.); u202112943@hust.edu.cn (R.W.); wysh@hust.edu.cn (Y.W.); cmj@hust.edu.cn (M.C.); hegy@hust.edu.cn (G.H.); ygx@hust.edu.cn (G.Y.);; 2 Grain Storage and Security Engineering Research Center of Education Ministry, School of Food and Strategic Reserves, Henan University of Technology, Zhengzhou 450052, China; limiao@haut.edu.cn; 3 College of Life Sciences and Health, Wuhan University of Science and Technology, Wuhan 430065, China

**Keywords:** tannic acid, commercial wheat flour, antioxidant activity, bread volume, crust and crumb color, sensory evaluation

## Abstract

Tannic acid (TA) has been recently considered as a new dough additive for improving the bread-making quality of wheat. However, the effects of TA supplementation on the sensory quality parameters (color, crumb grain structure, and sensory properties) of bread have not been studied. Further, the potential of TA supplementation in bread-making quality improvement has not been evaluated by using commercial flour. In the present study, three commercial wheat flours (namely, XL, QZG, and QZZ) with different gluten qualities were used to evaluate the effects of TA supplementation (in concentrations of 0.1% and 0.3%, respectively). TA supplementation did not change the proximate composition of the breads but increased the volumes and specific volumes of XL and QZG breads. TA supplementation enhanced antioxidant activities, with 0.3% TA significantly increasing the antioxidant capacities of bread made from all three flour samples by approximately four-fold (FRAP method)/three-fold (ABTS method). Positive effects of TA on the reduction in crumb hardness, gumminess, and chewiness were observed in the XL bread, as determined by the texture profile analysis. For the analyses on visual and sensory attributes, our results suggest that TA did not affect the crust color, but only slightly reduced the L* (lightness) and b* (yellowness) values of the crumb and increased the a* (redness) value. TA supplementation also increased the porosity, total cell area, and mean cell area. Satisfactorily, the sensory evaluation results demonstrate that TA-supplemented breads did not exhibit negative sensory attributes when compared to the non-TA-added breads; rather, the attributes were even increased. In summary, TA-supplemented breads generally had not only better baking quality attributes and enhanced antioxidant activities, but, more importantly, presented high consumer acceptance in multiple commercial flour samples. Our results support the commercial potential of TA to be used as a dough improver.

## 1. Introduction

White wheat bread is a major staple food in many countries and is also popular and consumed to varying degrees in other countries and regions of the world. Bread and other wheat flour products provide about 20% of the energy (calories) and 21% of plant-sourced proteins for human beings [1]. Moreover, wheat dough has unique viscoelasticity not possessed in other cereal flours, mainly due to gluten proteins [2]. Consequently, breads and other wheat-flour-derived food products hold both nutritional values and diverse culinary properties.

White flour represents a refined product of wheat grain. During the process of grain milling, the endosperm is separated from the germ and bran and refined to produce white flour [3]. Wheat brans and germs are removed during grain milling, which are rich in fiber (such as beta-glucan and arabinoxylan) and phytochemicals (such as sterols, phenols, vitamins and tocols) [4]. These compounds are thought to have positive effects on many health-related metabolic processes [5]. Consequently, the overconsumption of white flour and its food products may contribute to the increasing prevalence of chronic diseases (e.g., chronic kidney disease, cardiovascular diseases, neurodegenerative diseases, chronic obstructive pulmonary disease, and cancer) that are known to be associated with oxidative stress [6,7,8,9]. Accordingly, sensory quality and health benefits are increasingly important considerations for consumers when choosing breads and other bakery products [10]. Therefore, the development of innovative breads containing functional ingredients is a new important research area and echoes the consumers’ demands for healthy bakery products [11].

In addition, consumers expect bakery products to have a high sensory quality [12]. For bread, the volume and crumb grain structure are important quality parameters. Nevertheless, some studies have demonstrated that the addition of nutritious ingredients to bread, particularly those from coarse cereals enriched with nutrients, while improving the antioxidant capacity and/or the content of dietary fibers, significantly decreases the bread-making quality, therefore, impacting the appearance, texture or taste of the bread and consequently its acceptability [13,14,15,16]. For example, some polyphenols have also been reported to improve antioxidant capacity but reduce bread quality [17,18]. Therefore, it is essential to explore additives that enhance the antioxidant capacity without negatively affecting the sensory attributes of bread.

Tannins have been studied as potential fortifying ingredients for bread-making [19,20,21,22], as tannin compounds possess excellent antioxidant properties and are widely presented in many kinds of plants. Tannins can be separated into two groups following their distinct structural features: condensed and hydrolyzable tannins. Tannic acid (TA), a pale solid powder with a slightly astringent taste, is one typical compound of hydrolyzable tannins. TA consists of a central glucose unit and ten attached gallic acid molecules. TA is a safe food additive approved by the U.S. Food and Drug Administration (FDA) and the Joint FAO/WHO Expert Committee on Food Additives (JECFA). In the U.S.A., TA is a common additive widely used in beverages (wine, beer, and coffee) and pharmaceuticals, with an estimated daily use about 1 g of TA per person [23]. Previously, our group discovered that TA significantly improves the farinograph-based dough parameters [20]. Later, we confirmed that TA improves the dough quality by using a mixograph and identified that TA promotes glutenin aggregation and preferentially interacts with high-molecular-weight glutenin subunits (HMW-GS) through hydrogen bonds [21].

While the above-mentioned studies reveal the potential of TA as a new dough improver, key issues regarding the commercial application of TA in the baking industry remain to be addressed, and these include the following: (1) whether TA supplementation could affect sensory attributes and consumer acceptability of bread; (2) whether TA remains effective in bread quality improvement when supplemented into commercially used bread flour, since the previous studies examined TA’s positive effects in certain wheat cultivars that are grown locally (e.g., Zhengmai 9023) and not particularly bred for bread-making [20,21]. To address these issues, the present work aims to provide comprehensive analyses on several chemical and physical characteristics of the TA-supplemented bread made from commercial flour samples (including proximate compositions, baking parameters, antioxidant activity and bread texture), visual attributes (color analysis and crumb image analysis) and the sensory evaluation results.

## 2. Materials and Methods

### 2.1. Materials

Food-grade TA (Catalog No. 58454) was purchased from Henan Yiduoyuan Biotechnology Co., Ltd. (Zhengzhou, China). Three commercial wheat flours of different gluten strengths were purchased from the local market (Wuhan, China), including a high-gluten white bread flour Xinliang (XL), a bread flour Qizi (QZG) and a plain flour Qizi (QZZ). 

### 2.2. Preparation of Bread

Pan bread samples were made according to the AACC-approved method (AACC 10-10B) with slight modifications. TA was added at 0.1% and 0.3% of the dry weight of the wheat flour, respectively. Doughs were prepared at room temperature by mixing 40 g of wheat flour with 23.2 g of tap water, 0.4 g of dry yeast, 2.4 g of caster sugar, 0.6 g of table salt and 1.2 g of margarine. The baking temperatures and times were optimized for the above-mentioned scale. The dough was first fermented for 90 min in a controlled fermentation cabinet at 28 °C and 85% relative humidity. Then, the dough was formed into a cylindrical shape, placed in a baking mold (10 cm × 5.6 cm × 3.4 cm) and again had a second fermentation in the controlled fermentation cabinet for 60 min. Finally, the bread was baked for 20 min at 180 °C in an electric top-and-bottom heated oven. After cooling to room temperature, the bread was analyzed for loaf weight, loaf volume and specific volume. The baking experiment was performed in three batches.

### 2.3. Proximate Analysis

The fresh bread was used as a sample for moisture. The freeze-dried bread samples were analyzed to determine the ash, protein, fat and fiber. The moisture content was determined by the oven drying method according to 925.10 AOAC [24]. The ash content was performed by conductivity following ICC Standard No. 157 [25]. The protein content was determined by the Kjeldahl method using a KjeltecTM 8400 analyzer (Foss, Hilleroed, Denmark) following GB5009.5—2016. The nitrogen content was multiplied by 6.25 to obtain the protein content. The fat content was analyzed by hydrolysis in hydrochloric acid at 75 °C for 45 min followed by extraction in petroleum ether and evaporation according to GB5009.6—2016. The fiber content was evaluated using FIWE6 Fiber Analyzer (VELP, Usmate, Italy) according to GB5009.88—2014.

### 2.4. Volume and Specific Volume Measurement and Texture Profile

The loaf volume was determined according to the AACC-approved method 10-05 (rapeseed displacement test). The bread sample was placed in a measuring cylinder. The measuring cylinder was filled with rapeseed to the maximum line. The volume of the rapeseed after removing the bread sample was measured. The volume of the bread was calculated by subtracting the volume of rapeseed from the volume of the measuring cylinder. The specific volume was calculated by dividing the loaf volume by the loaf weight.

Three slices of bread including the middle slice and one on either side were used for texture profile analysis (TPA) at room temperature using a Texture Analyzer (TA-XT plus, Stable Micro Systems, Godalming, UK) according to a previous study [26]. The probe used was the P/36 aluminum cylinder (36.0 mm in diameter). The rates for the pre-test, test, and post-test were 1.0, 0.5, and 10.0 mm/s, respectively, the trigger force was 5.0 g, and the distance was 5.0 mm. The parameters analyzed were investigated in terms of their hardness, springiness, cohesiveness, gumminess, chewiness, and resilience.

### 2.5. Antioxidant Capacity Assays

A ferric-reducing antioxidant power assay (FRAP) kit (Catalog #G0115F) and a total antioxidant capacity assay kit with ABTS method (Catalog #G0142W) were purchased from Suzhou Grace Biotechnology Co., Ltd. (Suzhou, China). Antioxidant capacity was determined by the FRAP kit and ABTS kit. An amount of 10 mg of each of the freeze-dried bread samples were used for the FRAP assay and ABTS assay with three biological replicates. The results were expressed in µmol Trolox /g dry bread.

### 2.6. Determination of Crust and Crumb Color 

The color of bread crust and crumb was measured using a Minolta colorimeter (Minolta CR- 400, Konica Minolta Sensing, Inc., Osaka, Japan). Color values were recorded in ten areas of each loaf. The color analysis was expressed in L*, a*, and b* values. L* value is a measure of lightness (100: perfect white, zero: black), whereas a* (+red/−green) and b* (+yellow/−blue) values are the chromaticity values [27].

### 2.7. Image Analysis

Digital image analysis was used to evaluate crumb structure according to a previous study [28], as the image-analysis-based fineness score correlated well with the visual evaluation of fineness. The cross-sections of bread slices were scanned at 300 dpi on a grey scale with a scanner (CanoScan LiDE110, Tokyo, Japan). The images obtained were analyzed using ImageJ2 v1.0 software (National Institutes of Health, Bethesda, MD, USA). Sub-image areas of 30 mm × 30 mm were obtained at the same position on each slice (center of the bread) and then binarized for analysis. Five crumb features were determined, including the total number of cells, total area of cells, average area of cells, porosity, and percentage of the number of cells < 4 mm^2^.

### 2.8. Sensory Evaluation of Bread

A specially designed, odor-free meeting room with separate tables was kept at 22 ± 2 °C. The sensory evaluation was performed by a group of 20 evaluators (consumers) consisting of 11 males and 9 females, aged 18−45, all of whom are non-smokers. The experimental design of sensory evaluation was based on the sensory analyses of bread samples previously published in the Foods journal [29,30,31,32,33]. They were recruited from students and teachers working on the improvement of wheat processing quality at the Genetic Engineering International Cooperation Base of the Chinese Ministry of Science and Technology. The panelists are consumers of bread. To facilitate the description of the sensory characteristics of the bread, appearance, crumb characteristics, crust and crumb color, aroma, and taste were chosen based on previous report [14]. Appearance, crumb characteristics, crust and crumb color, aroma, and taste were evaluated using a 9-point Hedonic scale (1: dislike extremely, 5: neither like nor dislike, and 9: like extremely).

### 2.9. Statistical Analysis

All of the statistical analyses were conducted using the SPSS 26.0 software. All experiments were performed in triplicate. In order to determine the significance of differences between the flours, a two-way ANOVA was performed at a significance level of *p* < 0.05. The sensory data were checked for the homogeneity of variance and normality before performing ANOVA analysis with SPSS.

## 3. Results

### 3.1. Proximate Analysis

Three commercial wheat flours (namely XL, QZG and QZZ) were used in the present study. The proximate analysis of bread, measuring ash, protein, fat, and fiber, is shown in Table 1. Of the three flours, XL has the highest content of nutrients. QZZ has lower ash and protein content than QZG. The two-way statistical analysis showed a significant statistical effect (*p* < 0.05) of the type of flour on the composition of breads. As TA is a polyphenol, predictably, the addition of TA had no significant (*p* < 0.05) effect on the nutrient content of bread.

### 3.2. Baking Parameters

**Figure 1:**
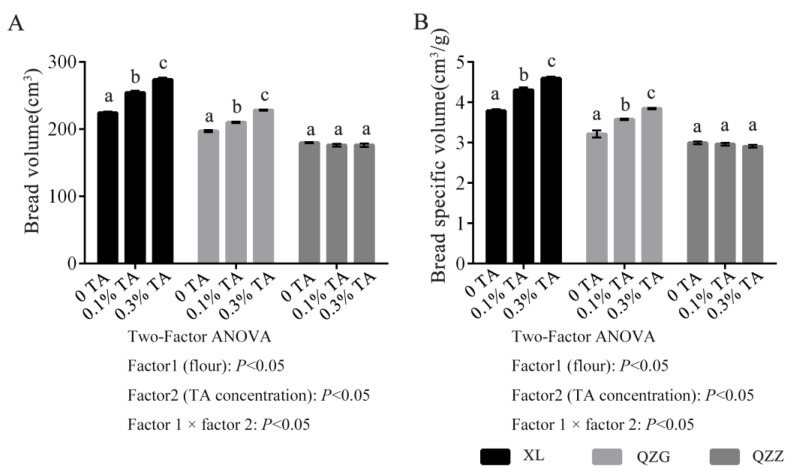
Effect of TA on baking characteristics of breads: bread volume (**A**) and bread specific volume (**B**). (**A**) The bread volume was determined according to the AACC-approved method (AACC 10-05); (**B**) The specific volume was calculated by dividing the bread volume by the bread weight. Different letters indicate values that are significantly different (*p* < 0.05, Tukey’s HSD test).

Next, we investigated whether TA addition to commercial flour could lead to improved dough quality. Without TA supplementation, the bread made from XL had the highest bread volume and specific volume, followed by QZG and QZZ (Figure 1A,B). TA supplementation showed different effects in improving bread volume. TA addition had no effect on QZZ breads, whereas TA supplementation significantly increased the bread volume and specific volume of XL and QZG breads in a dosage-dependent manner (from 0.1% to 0.3%). Indeed, TA improved the bread-making properties in commercial flour-made breads, and such improving effects became quite evident in high-gluten flour samples. The two-way statistical analysis showed a significant statistical effect (*p* < 0.05) of the type of flour on the baking parameters.

As a natural food additive, the effects of TA supplementation on bread-making properties are distinct from many other nutritional components. The addition of nut seeds, dietary fiber, polyphenols, plant extracts, or polyphenol-rich ingredients and vegetables all significantly reduced the bread volume [14,15,34,35,36]. The baking characteristics of the bread are correlated to the degree of crosslinking of gluten proteins and the addition of the above-mentioned ingredients likely led to weakened gluten networks, explaining why the bread-making quality was reduced. Unlike the above-mentioned supplements, TA has phenolic hydroxyl and gallic acyl groups that interact with gluten proteins via hydrogen bonding and hydrophobic interactions [20,21], leading to the dough containing well-distributed gluten polymers with a higher degree of cross-linking that could help improve the air retention ability of the dough during baking, thus improving the bread-making quality.

### 3.3. Antioxidant Activity of the TA-Supplemented Breads

The FRAP assay and ABTS assay were performed to test whether the TA-supplemented bread samples had enhanced antioxidant activity (Table 1). In the bread without TA, both FRAP and ABTS were about 4.5 μmol Trolox g^−1^ bread. TA supplementation dramatically increased the antioxidant capacity of the breads. The antioxidant capacity was increased by one-fold (approximately 10 μmol Trolox g^−1^ bread) in the FRAP assay and 0.5-fold in the ABTS assay (approximately 7.5 μmol Trolox g^−1^ bread) when supplemented with 0.1% TA and further increased by four-fold (~22 μmol Trolox g^−1^ bread) in the FRAP assay and three-fold in the ABTS assay (approximately 15 μmol Trolox g^−1^ bread) when supplemented with 0.3% TA. There was no statistical difference (*p* < 0.05) between the flours on the antioxidant activity according to the two-way ANOVA analysis.

Our results showed that the supplementation of low-level TA (i.e., 0.3%) can significantly increase the antioxidant activity of the bread samples. Previous studies have also reported improved antioxidant activity with the addition of nutritional ingredients. For instance, the addition of 10% sea buckthorn pulp, 8% flaxseed, and even 70% orange-fleshed sweet potato could increase the antioxidant activity of breads by two-fold, one-fold, and three-fold, respectively, as determined by the FRAP assay [34,37]. Although 6% apple polyphenols and 10% grape pomace powder (rich in polyphenols) improved the antioxidant capacity by about nine-fold and eight-fold, respectively [15,35], their amount of addition far exceeded the amount of TA added in our study. Previously, our work has shown that TA affects the dough’s macroscopic properties and microstructure through non-covalent interactions with gluten proteins, thus improving the dough processing parameters [21]. In the present study, TA supplementation improved not only the baking performance of breads (volume and specific volume of bread) (Figure 1A,B) but also the antioxidant capacity by around four-fold (FRAP assay)/three-fold (ABTS assay) (Table 1).

### 3.4. Texture Evaluation

The bread texture was evaluated after baking using TPA (Table 2). Without the addition of TA, XL bread had the least hardness, gumminess and chewiness among the three breads, followed by QZG and QZZ. After the addition of TA, TA significantly affected (*p* < 0.05) the hardness, gumminess and chewiness of XL bread, while there was no significant effect (*p* < 0.05) on the other parameters. TA did not produce a significant effect (*p* < 0.05) on the TPA parameters of either QZG or QZZ. The two-way statistical analysis showed a significant statistical effect (*p* < 0.05) by the type of flour on the hardness, cohesiveness, gumminess and chewiness.

Texture properties determine product durability and consumer acceptance. A denser crumb structure leads to greater hardness [34]. To maximize consumer acceptance of the bread, minimizing the hardness value is the highest priority [38]. The effect of TA on the hardness of bread meets this requirement, which is reflected in the fluffier crumb structure of TA-added bread. The dextran produced by the sourdough fermentation of whole and sprouted lentil flour affects the crumb structure of white bread, resulting in less hardness [39]. Additionally, chewiness is a dimensionless size and characterizes the energy needed to chew the food [34]. A positive effect of TA on the reduction in adhesion and chewiness of bread crumb was observed, which may be attributed to the effect of TA on the gluten protein network. 

### 3.5. Visual Attributes of the TA-Supplemented Breads

The visual attributes analyzed in the present study include crumb color, crust color and crumb structure parameters [40].

#### 3.5.1. Color Analysis

**Figure 2:**
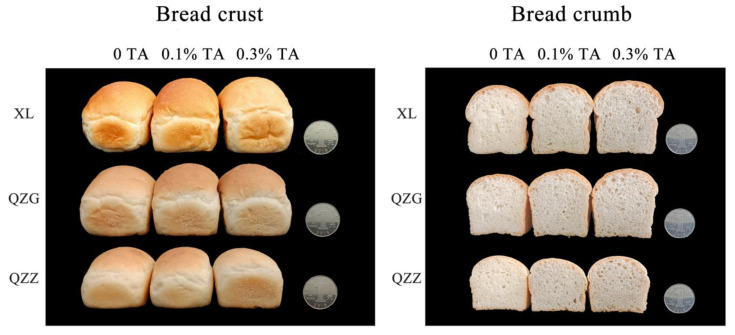
Effect of TA on crust and crumb color.

Since TA is a pale-yellow powder, we also investigated the crumb color of TA-supplemented bread samples. Visual comparison did not reveal obvious changes in the crumb color between the bread slices supplemented with or without TA (Figure 2). The crust colors of the bread samples were then analyzed by using a colorimeter (Table 3). In the non-TA-added crust samples, the L* and b* values of the crust color were different between the flour samples, ranking from high to low as QZZ, QZG, and XL, while no difference was detected for the a* value. After TA supplementation, a slight increase in L* values for QZZ and XL bread crusts and a slight decrease for QZG was detected. TA supplementation led to a slight decrease in b* values of the QZZ and QZG bread crusts but a slight increase in the XL bread crust. Generally, TA addition did not affect a* values.

Although a two-way ANOVA analysis showed significant differences (*p* < 0.05) in color between flours, the differences in crumb color values were not large between the three bread samples (i.e., XL, QZG and QZZ). After TA addition, as the concentration of TA increased, the crumb L* and b* values gradually decreased in all three breads, while the crumb a* values increased gradually. Overall, the crust colors were not changed significantly by TA supplementation, and the crumb colors became slightly deepened based on colorimeter analysis.

Previous studies have shown that bread color can be influenced by additives in a dosage-dependent manner [13,35,41]. When millet flour was added to the bread at 10%, 30% and 50% percent, respectively, the crumb color darkened as the amount of millet flour increased [13]. Another example is that gluten-free bread made with pigmented rice flour (containing 19% anthocyanins) shows a purple color [41]. Here, color analysis results showed that the effect of TA on crumb color values was dosage-dependent (Table 3). Because TA is only pale yellow in color and its effective amount of addition is pretty low (0.3%), TA supplementation into breads only leads to a subtle (albeit detectable for some parameters by the colorimeter) change in crust color values and may not affect consumer acceptability.

#### 3.5.2. Crumb Image Analysis

**Figure 3:**
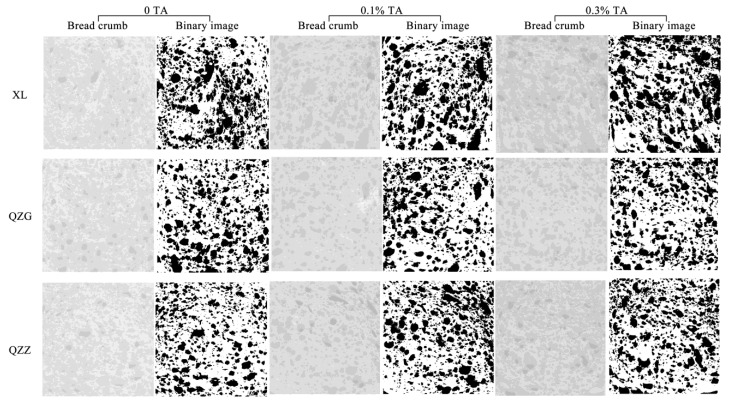
Determination of the visual attributes of bread samples with or without TA supplementation. Cross-section of bread crumb and its binary image with different levels of TA. The binary image was obtained by analyzing a 30 mm × 30 mm bread crumb area with ImageJ2 software (National Institutes of Health, USA).

Flour additives can influence the crumb characteristics of bread, which are related to the end-use quality of the bread [42]. To obtain a detailed and quantitative evaluation of the crumb structure, image analysis was performed on slices of breads supplemented with/without TA (Figure 3). The total area of cells, average area of cells, and porosity were correlated with the bread volume and specific volume, and the percentage of total cells and those of the cells < 4 mm^2^ were correlated with the gas–cell regularity (Figure 3) [43].

The two-way statistical analysis showed a significant statistical effect (*p* < 0.05) of the type of flour on the gas cell characteristic. Without TA addition, the porosity, total area of cells, and average area of cells were highest in the XL bread, followed by the QZG and QZZ bread (Table 4). Although the total number of cells of the QZG bread was lower than that of the QZZ bread, the total area of cells, average area of cells, and porosity of the QZG bread was higher than that of the QZZ bread. Theoretically, large values of the average area of cells, porosity, and total area of cells imply high bread volumes [44]. Our results of crumb structure parameters were consistent with the bread volume results (Figure 1). Overall, TA supplementation significantly altered crumb structures, and those changes were reflected in the image analysis results. TA supplementation significantly increased (*p* < 0.05) the total area of cells, average area of cells, and porosity of the XL breads, while TA addition to QZG breads led to a significantly increased (*p* < 0.05) total area of cells and porosity. Further, TA supplementation into the QZZ breads resulted in significantly increased (*p* < 0.05) total area of cells, average area of cells, and porosity compared to those of non-TA breads. However, the percentage of the number of cells < 4 mm^2^ of the XL or QZG breads decreased significantly along with the increased TA dosage. These results indicate that TA addition increased the number of large cells, while we did not detect statistical differences in the total number of cells with or without TA supplementation. In summary, TA supplementation improved the crumb structure parameters. Moreover, TA addition had more pronounced effects on the XL bread than those made from QZG and QZZ flour samples.

TA supplementation increased the total area of cells, average area of cells, and porosity, consistent with the improved bread volume and specific volume of the TA-added breads. According to a previous report, the addition of barley β-glucan to the bread could increase the total and average area of cells, while the specific volume of the bread became larger [43]. By contrast, the addition of 10–14% soluble oat fiber decreased the specific volume of the bread by 50% compared to the control bread, and the total area of cells and porosity were decreased as well [45].

The total number of cells in a slice of bread serves as an indication of the number of carbon dioxide bubbles captured during proofing [46]. TA addition did not significantly affect the total number of cells compared to the control, indicating that TA addition may not affect the ability to capture carbon dioxide bubbles during proofing. Previous studies showed that bread containing 4–12% of apricot kernel flour does not allow gas cells to expand, resulting in smaller gas chambers in the bread and thus a compact crumb, consistent with the fact that apricot kernel flour is less elastic than gluten proteins [44]. Unlike the undesirable effects brought by apricot kernel flour, TA addition indeed enhanced the dough property to allow CO_2_ to expand properly, resulting in a greater porosity.

### 3.6. Effect of TA Supplementation on the Bread Sensory Attributes 

In the sensory evaluation experiments, the six sensory attributes were scored by the semi-trained panelists (Table 5). A two-way ANOVA analysis showed significant differences (*p* < 0.05) in crumb characteristics and taste between breads. The XL bread without TA addition received the highest score in the categories of crumb characteristics and taste, which might be associated with the highest specific volume of non-TA XL bread among all the bread samples. When compared with the non-TA bread samples, only TA-added XL bread samples obtained significantly higher scores for several sensory attributes, while in the other two breads (QZG and QZZ), TA addition did not differ from the control. The score of textural attributes for XL was significantly increased (*p* < 0.05) at the 0.1% TA addition level compared to the control. When TA was added at 0.3%, the appearance, crumb characteristics, taste, and crust color of XL were significantly increased (*p* < 0.05) compared to the bread without TA addition. This indicated that the TA-added XL breads were more popular. It was found that both appearances decreased when gas cells were smaller and evenly distributed [41]. TA addition led to larger and uneven gas cells in the XL breads, thus producing an improved loaf appearance. Crumb color is an important sensory attribute that influences consumer preferences. For the breads made from all three types of flour, the crumb color scores of the breads added with 0.1% or 0.3% TA did not differ significantly compared to the breads without TA, which was consistent with the colorimeter results (Table 3). Therefore, both results suggest that TA supplementation into breads did not reduce sensory attributes and consumer acceptability.

Previous studies have shown that the bread taste changes as the amount of natural ingredients added increases [47,48,49]. For example, 0.5% or more microencapsulated onion skin extracts made the bread taste obviously bitter compared to those added with low concentrations of the onion skin extracts [48]. The addition of 15% prickly pear peel flour made the bread sour while the addition of 20% changed the bitterness and astringency of the bread [47]. At 4% and higher additions of dandelion root, the bread could be perceived to have an unusual odor and an obvious bitterness [49]. The TA in this study did not change the aroma of the bread as it was added at a mere 0.3%.

## 4. Conclusions

Our research demonstrates that the addition of TA to commercial high-gluten flours produces breads with greater volume and specific volume. The addition of 0–0.3% TA to commercial flour did not affect the proximate composition of the bread. Importantly, TA addition into bread is likely an effective way to produce breads with high antioxidant activity. The addition of TA to commercial high-gluten bread flour had a lower hardness, gumminess and chewiness. TA supplementation, at least at the concentrations tested herein, did not negatively affect bread color and appearance. Moreover, the sensory characteristics of TA-supplemented bread (made from the high-gluten bread flour) were scored the highest for the appearance, crumb characteristics, taste and crust color in our sensory evaluation. Overall, our study demonstrated that TA represents as an effective bread additive for the development of breads with bioactive compounds and increased antioxidant activity and the quality of the bread and acceptable sensory quality.

## Figures and Tables

**Table 1:** Proximate value and antioxidant activity of breads supplemented with or without TA.

Breads	Samples	Ash Content (g/100 g DM)	Protein Content (g/100 g DM)	Fat Content (g/100 g DM)	Fiber Content (g/100 g DM)	ABTS (µmol TE/g DM)	FRAP (µmol TE/g DM)
XL	0	6.17 ± 0.14 ^a^	13.74 ± 0.09 ^a^	3.23 ± 0.10 ^a^	3.23 ± 0.10 ^a^	4.65 ± 0.75 ^a^	4.27 ± 0.34 ^a^
	0.10%	6.02 ± 0.05 ^a^	13.76 ± 0.07 ^a^	3.46 ± 0.09 ^a^	3.46 ± 0.09 ^a^	10.11 ± 0.88 ^b^	9.58 ± 0.05 ^b^
	0.30%	6.10 ± 0.08 ^a^	13.44 ± 0.18 ^a^	3.52 ± 0.03 ^a^	3.59 ± 0.04 ^a^	15.03 ± 1.51 ^c^	21.97 ± 0.08 ^c^
QZG	0	5.85 ± 0.06 ^b^	11.05 ± 0.06 ^a^	2.94 ± 0.04 ^a^	2.94 ± 0.04 ^a^	4.14 ± 0.07 ^a^	4.24 ± 0.08 ^a^
	0.10%	5.97 ± 0.09 ^b^	11.07 ± 0.02 ^a^	2.97 ± 0.08 ^a^	2.97 ± 0.08 ^a^	7.34 ± 0.21 ^b^	10.09 ± 0.16 ^b^
	0.30%	5.37 ± 0.16 ^a^	11.21 ± 0.08 ^a^	2.91 ± 0.10 ^a^	2.91 ± 0.10 ^a^	16.71 ± 0.54 ^c^	22.30 ± 0.11 ^c^
QZZ	0	5.36 ± 0.08 ^a^	10.28 ± 0.10 ^a^	3.02 ± 0.14 ^a^	3.02 ± 0.14 ^a^	4.43 ± 0.74 ^a^	3.97 ± 0.13 ^a^
	0.10%	5.19 ± 0.08 ^a^	10.41 ± 0.09 ^a^	2.88 ± 0.03 ^a^	2.88 ± 0.03 ^a^	6.68 ± 0.22 ^a^	10.15 ± 0.20 ^b^
	0.30%	5.52 ± 0.09 ^a^	10.46 ± 0.07 ^a^	2.68 ± 0.08 ^a^	2.68 ± 0.08 ^a^	15.52 ± 1.49 ^b^	22.57 ± 0.17 ^c^
Two-Factor ANOVA—*p*							
Factor 1 (flour)		<0.05	<0.05	<0.05	>0.05	>0.05	>0.05
Factor 2 (TA concentration)		>0.05	>0.05	>0.05	<0.05	<0.05	<0.05
Factor 1 × factor 2		<0.05	>0.05	<0.05	>0.05	>0.05	>0.05

Lower case letters indicate the statistical results of a bread proximate component or antioxidant activity (corresponding to each of the columns) between control and TA treatments. Different letters indicate values that are significantly different (*p* < 0.05, Tukey’s HSD test).

**Table 2:** Textural parameters of breads supplemented with or without TA.

Breads	Samples	Hardness (N)	Springiness (−)	Cohesiveness (−)	Gumminess (−)	Chewiness (−)	Resilience (−)
XL	0 TA	950.75 ± 9.11 ^c^	0.95 ± 0.01 ^a^	0.74 ± 0.01 ^a^	705.87 ± 9.32 ^b^	672.74 ± 9.79 ^b^	0.43 ± 0.01 ^a^
	0.1% TA	858.99 ± 14.28 ^b^	0.97 ± 0.02 ^a^	0.78 ± 0.03 ^a^	667.46 ± 16.42 ^b^	645.86 ± 25.17 ^b^	0.47 ± 0.03 ^a^
	0.3% TA	595.73 ± 7.14 ^a^	0.96 ± 0.01 ^a^	0.78 ± 0.02 ^a^	464.50 ± 8.30 ^a^	447.60 ± 11.50 ^a^	0.47 ± 0.01 ^a^
QZG	0 TA	1243.19 ± 32.00 ^ab^	0.97 ± 0.01 ^a^	0.68 ± 0.01 ^a^	840.05 ± 15.14 ^a^	814.07 ± 16.15 ^a^	0.40 ± 0.01 ^a^
	0.1% TA	1272.64 ± 9.04 ^b^	0.96 ± 0.01 ^a^	0.74 ± 0.03 ^a^	934.70 ± 31.12 ^a^	896.29 ± 22.03 ^b^	0.43 ± 0.02 ^a^
	0.3% TA	1143.08 ± 38.36 ^a^	0.97 ± 0.01 ^a^	0.74 ± 0.01 ^a^	840.94 ± 16.66 ^a^	812.32 ± 15.91 ^a^	0.45 ± 0.01 ^a^
QZZ	0 TA	2359.12 ± 88.59 ^a^	0.97 ± 0.01 ^a^	0.71 ± 0.06 ^a^	1653.40 ± 77.11 ^a^	1610.66 ± 75.72 ^a^	0.45 ± 0.06 ^a^
	0.1% TA	2221.69 ± 95.21 ^a^	0.98 ± 0.01 ^a^	0.67 ± 0.01 ^a^	1495.30 ± 60.60 ^a^	1459.18 ± 61.83 ^a^	0.42 ± 0.01 ^a^
	0.3% TA	2167.52 ± 107.32 ^a^	0.97 ± 0.01 ^a^	0.66 ± 0.03 ^a^	1414.10 ± 21.97 ^a^	1371.37 ± 10.89 ^a^	0.40 ± 0.03 ^a^
Two-Factor ANOVA—*p*							
Factor 1 (flour)		<0.05	>0.05	<0.05	<0.05	<0.05	>0.05
Factor 2 (TA concentration)		<0.05	>0.05	>0.05	<0.05	<0.05	>0.05
Factor 1 × factor 2		>0.05	>0.05	>0.05	<0.05	<0.05	>0.05

Lower case letters indicate the statistical results of a textural parameter (corresponding to each of the columns) between control and TA treatments. Different letters indicate values that are significantly different (*p* < 0.05, Tukey’s HSD test).

**Table 3:** The crust and crumb color parameters of the bread samples supplemented with or without TA.

Breads	Samples	Crust Color			Crumb Color		
		L*	a*	b*	L*	a*	b*
XL	0 TA	68.31 ± 0.75 ^a^	16.98 ± 0.53 ^ab^	29.91 ± 0.98 ^a^	90.64 ± 0.24 ^a^	0.71 ± 0.03 ^a^	10.58 ± 0.18 ^a^
	0.1% TA	70.77 ± 0.54 ^b^	17.85 ± 0.35 ^b^	34.21 ± 0.49 ^b^	83.55 ± 0.27 ^b^	2.05 ± 0.01 ^b^	4.98 ± 0.15 ^b^
	0.3% TA	72.99 ± 0.22 ^c^	15.71 ± 0.24 ^a^	34.40 ± 0.42 ^b^	82.17 ± 0.19 ^c^	3.03 ± 0.01 ^c^	5.32 ± 0.09 ^b^
QZG	0 TA	79.91 ± 0.80 ^a^	18.68 ± 0.27 ^a^	39.87 ± 0.50 ^a^	90.90 ± 0.21 ^a^	0.98 ± 0.02 ^a^	10.09 ± 0.24 ^a^
	0.1% TA	78.79 ± 0.18 ^a^	17.37 ± 0.87 ^ab^	37.77 ± 0.61 ^a^	85.41 ± 0.15 ^b^	1.95 ± 0.02 ^b^	7.13 ± 0.11 ^b^
	0.3% TA	74.57 ± 0.89 ^b^	16.40 ± 0.38 ^b^	34.54 ± 0.78 ^b^	84.01 ± 0.25 ^c^	2.79 ± 0.01 ^c^	6.84 ± 0.21 ^b^
QZZ	0 TA	83.75 ± 0.89 ^ab^	16.78 ± 0.41 ^ab^	42.10 ± 1.02 ^a^	85.94 ± 0.17 ^a^	0.97 ± 0.02 ^a^	11.00 ± 0.24 ^a^
	0.1% TA	82.36 ± 1.50 ^b^	19.01 ± 1.30 ^a^	42.11 ± 0.34 ^a^	81.71 ± 0.26 ^b^	1.75 ± 0.03 ^b^	7.68 ± 0.24 ^b^
	0.3% TA	86.36 ± 0.77 ^a^	13.99 ± 0.66 ^b^	39.47 ± 0.37 ^b^	78.75 ± 0.19 ^c^	2.74 ± 0.02 ^c^	8.63 ± 0.17 ^c^
Two-Factor ANOVA—*p*							
Factor1 (flour)		<0.05	>0.05	<0.05	<0.05	<0.05	<0.05
Factor 2 (TA concentration)		<0.05	<0.05	<0.05	<0.05	<0.05	<0.05
Factor 1 × factor 2		>0.05	<0.05	<0.05	<0.05	<0.05	<0.05

Lower case letters indicate the statistical results of a crust or crumb color parameter (corresponding to each of the columns) between control and TA treatments. Different letters indicate values that are significantly different (*p* < 0.05, Tukey’s HSD test).

**Table 4:** The gas cell characteristics of the bread samples supplemented with or without TA.

Breads	Samples	Total Number of Cells	Total Area of Cells (mm^2^)	Average Area of Cells (mm^2^)	Porosity (%)	Percentage of Number of Cells < 4 mm^2^ (%)
XL	0 TA	1419.33 ± 43.43 ^a^	323.07 ± 16.04 ^a^	0.23 ± 0.02 ^a^	35.23 ± 1.57 ^a^	99.00 ± 0.15 ^a^
	0.1% TA	1242.33 ± 65.58 ^a^	353.30 ± 10.23 ^a^	0.29 ± 0.02 ^ab^	37.77 ± 1.14 ^a^	98.43 ± 0.16 ^ab^
	0.3% TA	1322.33 ± 18.17 ^a^	411.43 ± 2.91 ^b^	0.31 ± 0.00 ^b^	43.38 ± 0.70 ^b^	98.33 ± 0.12 ^b^
QZG	0 TA	1223.00 ± 39.51 ^a^	262.60 ± 14.88 ^a^	0.22 ± 0.02 ^a^	28.54 ± 1.52 ^a^	99.10 ±0.13 ^a^
	0.1% TA	1290.33 ± 52.60 ^a^	278.55 ± 2.01 ^ab^	0.22 ± 0.01 ^a^	30.32 ± 0.49 ^ab^	99.10 ± 0.01 ^a^
	0.3% TA	1184.33 ± 20.76 ^a^	313.25 ± 4.44 ^b^	0.26 ± 0.01 ^a^	33.87 ± 0.59 ^b^	98.42 ± 0.23 ^b^
QZZ	0 TA	1492.33 ± 6.39 ^a^	253.38 ± 14.45 ^a^	0.17 ± 0.01 ^a^	27.22 ± 1.41 ^a^	99.35 ± 0.18 ^a^
	0.1% TA	1412.67 ± 28.90 ^a^	308.20 ± 10.97 ^b^	0.22 ± 0.01 ^b^	33.60 ± 1.31 ^b^	99.05 ± 0.04 ^a^
	0.3% TA	1498.67 ± 37.68 ^a^	309.83 ± 5.51 ^b^	0.21 ± 0.01 ^b^	33.32 ± 0.64 ^b^	99.09 ± 0.13 ^a^
Two-Factor ANOVA—*p*						
Factor1 (flour)		<0.05	<0.05	<0.05	<0.05	<0.05
Factor2 (TA concentration)		>0.05	<0.05	<0.05	<0.05	<0.05
Factor 1 × factor 2		<0.05	>0.05	>0.05	>0.05	>0.05

Lower-case letters indicate the statistical results of a gas cell characteristic (corresponding to each of the columns) between the control and tannic acid treatments (*p* < 0.05, Tukey’s HSD test).

**Table 5:** The sensory attributes of the bread samples supplemented with or without TA.

Breads	Samples	Appearance	Crumb Characteristics	Crust Color	Crumb Color	Aroma	Taste
XL	0 TA	7.07 ± 0.18 ^a^	7.27 ± 0.40 ^a^	7.00 ± 0.26 ^a^	7.47 ± 0.24 ^a^	7.60 ± 0.27 ^a^	7.53 ± 0.26 ^a^
	0.1% TA	7.20 ± 0.24 ^a^	8.13 ± 0.19 ^b^	7.47 ± 0.22 ^a^	7.67 ± 0.21 ^a^	7.80 ± 0.22 ^a^	8.13 ± 0.19 ^a^
	0.3% TA	7.80 ± 0.22 ^b^	8.60 ± 0.19 ^c^	7.67 ± 0.21 ^b^	7.80 ± 0.35 ^a^	7.73 ± 0.25 ^a^	8.40 ± 0.34 ^b^
QZG	0 TA	7.27 ± 0.21 ^a^	7.13 ± 0.24 ^a^	7.00 ± 0.24 ^a^	7.33 ± 0.27 ^a^	7.27 ± 0.32 ^a^	6.33 ± 0.37 ^a^
	0.1% TA	7.73 ± 0.21 ^a^	7.40 ± 0.25 ^a^	7.33 ± 0.23 ^a^	7.47 ± 0.24 ^a^	7.67 ± 0.23 ^a^	6.63 ± 0.41 ^a^
	0.3% TA	7.60 ± 0.16 ^a^	7.60 ± 0.21 ^a^	7.73 ± 0.23 ^b^	7.33 ± 0.23 ^a^	7.40 ± 0.31 ^a^	7.03 ± 0.23 ^a^
QZZ	0 TA	7.27 ± 0.28 ^a^	6.53 ± 0.32 ^a^	7.27 ± 0.28 ^a^	7.27 ± 0.18 ^a^	7.60 ± 0.16 ^a^	6.27 ± 0.33 ^a^
	0.1% TA	7.40 ± 0.31 ^a^	7.13 ± 0.17 ^a^	7.40 ± 0.24 ^a^	7.07 ± 0.30 ^a^	7.13 ± 0.35 ^a^	6.93 ± 0.28 ^a^
	0.3% TA	7.53 ± 0.31 ^a^	7.07 ± 0.27 ^a^	7.67 ± 0.21 ^a^	7.20 ± 0.24 ^a^	6.93 ± 0.45 ^a^	7.00 ± 0.26 ^a^
Two-Factor ANOVA—*p*							
Factor1 (flour)		>0.05	<0.05	>0.05	>0.05	>0.05	<0.05
Factor2 (TA concentration)		>0.05	<0.05	<0.05	>0.05	>0.05	<0.05
Factor 1 × factor 2		>0.05	>0.05	>0.05	>0.05	>0.05	>0.05

Lower-case letters indicate the statistical analysis of a sensory attribute (corresponding to each of the columns) between the control and tannic acid treatments (*p* < 0.05, Tukey’s HSD test).

## Data Availability

Data is contained within the article.
